# Preparation and in vitro evaluation of photodynamic-responsive nanoliposome loaded PL-5

**DOI:** 10.1371/journal.pone.0351679

**Published:** 2026-06-16

**Authors:** Wen Lin, Qiong-zhi Shi, Xiang-ru Liao, Yuan Zeng, Xiang-yang Xie, Gang-jian Ji, Yin-ke Li

**Affiliations:** 1 Department of Clinical Laboratory, Wuhan Third Hospital (Tongren Hospital of Wuhan University), Wuhan, China; 2 Department of Pharmacy, General Hospital of Central Theater Command, Wuhan, China; 3 College of Nursing and Health Management and College of Life Science and Chemistry, Wuhan Donghu University, Wuhan, China; 4 Department of Pharmacy, Wuhan Third Hospital (Tongren Hospital of Wuhan University), Wuhan, China; Mass Eye Infirmary, Harvard Medical School / Northeastern University, UNITED STATES OF AMERICA

## Abstract

Burn wound infections are frequently complicated by biofilm-forming and multidrug-resistant pathogens, particularly methicillin-resistant Staphylococcus aureus (MRSA), posing major therapeutic challenges. Antimicrobial peptides (AMPs) such as PL-5 (peceleganan) exhibit broad-spectrum activity but are limited by instability, poor biofilm penetration, and reduced efficacy in complex wound environments. Here, a red-light-responsive, porphyrin-phospholipid (PoP)-containing cationic liposomal system for PL-5, aiming to enhance its antibacterial and antibiofilm performance was developed. Optimized liposomes achieved high encapsulation efficiency (~73%), uniform nanoscale size (~50 nm), narrow polydispersity, and positive surface charge. They demonstrated good storage stability and controlled peptide release under red-light irradiation (635 nm). *In vitro*, red-light activation significantly enhanced antimicrobial activity against MRSA and methicillin-susceptible *S. aureus* (MSSA), reducing minimum inhibitory concentration (MIC) values fourfold and accelerating bactericidal kinetics compared with free PL-5 and non-irradiated liposomes. Additionally, red-light-activated liposomes markedly inhibited biofilm formation. These results indicate that light-responsive liposomal delivery enables spatiotemporally controlled release of PL-5, significantly potentiating its antibacterial and antibiofilm efficacy. This approach offers a promising localized treatment strategy for biofilm-associated burn wound infections and a foundation for future translational studies.

## 1. Introduction

Burn wound infections remain a significant clinical and therapeutic challenge worldwide, with microbial complications accounting for up to 75% of deaths in severe burn injuries [1]. Among the pathogens colonizing burn wounds, *Staphylococcus aureus* is consistently the most prevalent [2,3]. Methicillin-resistant *S. aureus* (MRSA) represents a particularly severe threat due to its extensive antimicrobial resistance, biofilm-forming capacity, and ability to cause recurring outbreaks [4,5]. The organism is highly adaptable, colonizing nares, skin, and mucosal surfaces [6] and expressing multiple virulence factors—including α-toxin, Panton-Valentine leukocidin, and superantigens—that promote tissue invasion, immune evasion, and toxin-mediated shock [7,8]. Traditional treatments rely on β-lactams (including penicillins and cephalosporins), aminoglycosides, tetracyclines, and chloramphenicol; however, widespread resistance has greatly reduced their efficacy [9]. MRSA strains harbor altered penicillin-binding proteins (PBPs), rendering β-lactams ineffective [10]. Glycopeptides, such as vancomycin, remain a last-line therapy, yet reports of reduced susceptibility and emerging VRSA strains further compromise treatment options [11]. Additionally, *S. aureus* can metabolically adapt to the wound microenvironment, increasing tolerance to antibiotics and host immunity [12].

Biofilm formation exacerbates the clinical severity of burn infections. Biofilm-associated *S. aureus* exhibits up to 1,000-fold increased tolerance to antibiotics and contributes to persistent wound colonization [13,14]. Biofilms limit antimicrobial penetration, shield bacteria from host defenses, and facilitate chronic infection cycles [15,16]. Consequently, alternative therapeutic approaches targeting biofilms are urgently needed.

Given these multifactorial defense mechanisms, novel approaches such as bacteriophage therapy, antimicrobial peptides (AMPs), and nanotechnology-based drug delivery systems are actively being explored to overcome bacterial resistance and improve treatment outcomes [17,18]. AMPs are particularly promising due to their membrane-disruptive mechanism and low likelihood of resistance development [19,20]. PL-5 (peceleganan), a 26-residue α-helical AMP, is the first topical peptide approved for clinical trials in skin infections, demonstrating significant wound healing acceleration and infection control [21]. However, as a peptide, its clinical application may be limited by rapid proteolytic degradation, short residence time, inadequate penetration through biofilms, and dose-dependent cytotoxicity in free form [20].

Nanotechnology offers solutions for overcoming enzymatic degradation, improving delivery efficiency, and enhancing biofilm penetration [22]. Among nanocarriers, liposomes are particularly attractive due to their capacity for controlled release, protection of peptides, biofilm matrix‐/cell membrane‐fusogenicity and preferential accumulation in infected tissues [23,24]. Stimuli-responsive nanocarriers—triggered by pH, enzymes, temperature, or light—enable localized, on-demand drug release, minimizing systemic exposure [25]. In particular, light-responsive liposomes offer unique advantages: the external application of light provides precise spatiotemporal control of drug release, allowing burst delivery of antimicrobial agents directly at the infection site while preserving surrounding healthy tissue. Such light-controlled release can be achieved using clinical devices such as low-power red-light LED arrays, handheld laser systems, or fiber-optic-based illumination, allowing clinicians to adjust intensity, duration, and wavelength to match the depth and size of the wound [26]. These approaches may increase local AMP concentrations at infected wound sites while limiting systemic exposure. The use of wearable or portable light-emitting devices could enable repeated or sustained photo-triggered activation on burn wounds in outpatient settings. In addition, the spatial control provided by light activation may allow localized drug release within heterogeneous wound areas, enhancing activity against dense biofilms while minimizing effects on surrounding healthy tissue [26]. Overall, light-triggered delivery offers greater spatial and temporal control than conventional systemic or topical therapies that rely on passive diffusion.

In this study, PL-5 was chosen as the model drug, and photodynamic-responsive (PoP-containing) [27] cationic liposomes were designed and prepared. The formulation aims to enhance the stability of PL-5 in physiological environments, improve penetration into biofilm matrices, enable red-light-triggered release at the site of infection, and augment antimicrobial and antibiofilm activity against MRSA and MSSA. Upon red-light irradiation, the liposomal membrane becomes permeabilized, facilitating the controlled release of PL-5. Sustained release under continued illumination ensures prolonged antibacterial efficacy and allows flexible modulation of dosing based on wound severity and clinician preference. To achieve these goals, we optimized the liposomal formulation using response surface methodology (RSM) and conducted comprehensive physicochemical characterization, stability assessment, photo-triggered release behavior, and evaluation of antibacterial and antibiofilm performance.

By establishing a robust, reproducible platform for light-responsive AMP delivery, this work lays the groundwork for future translational studies. These will include preclinical evaluation in infected burn wound animal models, comprehensive safety and toxicity assessments, and the development of clinically compatible light-delivery systems for localized therapeutic activation. Ultimately, these studies aim to improve the clinical management of biofilm-associated burn wound infections, highlighting the potential of light-controlled drug delivery as a versatile and highly precise therapeutic strategy.

## 2. Materials and methods

### 2.1. Materials

The antimicrobial peptide PL-5 (Ac-KWKSFLKTFKSAAKTVLHTALKAISS-NH_2_, MW: 2933.5 Da, > 98% purity) was provided by Abmole (Shanghai, China). Cholesterol (Chol), 1,2-distearoyl-sn-glycero-3-phosphocholine (DSPC) and 1,2-distearoyl-sn-glycero-3-phosphoethanolamine-N-[poly(ethylene glycol)-_2000_] (DSPE-PEG_2000_), all of which were obtained from Shanghai Macklin Biochemical Co., Ltd (Shanghai, China). 1,2-dioleoyl-3-trimethylammonium-propane (DOTAP) was provided by Aladdin Scientific Co., Ltd (Shanghai, China). All other chemicals and solvents were purchased from Sinopharm Chemical Reagent Co., Ltd. (Shanghai, China) and were of analytical or high-performance liquid chromatography (HPLC) grade.

Methicillin-resistant *S. aureus* (MRSA, ATCC43300) and methicillin-susceptible *S. aureus* (MSSA, ATCC25923) were purchased from Biofeng Reagent Co., Ltd. (China). All cultures were routinely cultured in Mueller-Hinton Broth (MHB) and maintained on Mueller-Hinton Agar (MHA). Bacterial cultures were incubated at 37°C in an atmosphere supplemented with 5% CO_2_ to ensure optimal growth conditions.

### 2.2. Preparation of liposomes

Porphyrin-phospholipid (PoP) were synthesized as previously reported [27]. The liposomal formulations were prepared by a thin-film hydration method. Different amounts of DSPC, cholesterol, PoP, DOTAP and DSPE-PEG_2000_ (Table 1) were dissolved in ethanol (2:1, v/v) in a round-bottom flask. The lipid solution was evaporated under reduced pressure at 50°C using a rotary evaporator until a thin lipid film was formed on the inner wall of the flask. The thin lipid film was hydrated with 20 mL of PL-5 (2 mg/mL in PBS, pH 7.4) at 60°C for 1 hour to form multilamellar vesicles (MLVs). The PL-5 solution was prepared in PBS (pH 7.4). To obtain liposomes of uniform size, the multilamellar vesicles (MLVs) were first processed using an AH100D high-pressure homogenizer (ATS Engineering Inc., Canada) for preliminary size reduction. The suspension was then extruded 10 times through a polycarbonate membrane filter (pore size: 50 nm) using a hand-held extruder (e.g., Avanti Polar Lipids) at 60°C to ensure uniformity and a narrow size distribution. All liposomal formulations were prepared in three independent batches, and each batch was characterized independently to confirm reproducibility.

**Table 1:** Three-level factorial response surface design layout and observed responses for liposomes loaded with PL-5.

Run	Batch codes	Coded X_1_	Coded X_2_	Actual X_1_	Actual X_2_	Response Y (EE, %)
9	F1	−1	−1	1	0.5	58.5
1	F2	0	−1	2	0.5	67.4
2	F3	1	−1	3	0.5	60.4
11	F4	−1	0	1	1	62.3
6	F5*	0	0	2	1	70.3
3	F5	1	0	3	1	64.9
4	F5	−1	1	1	1.5	65.1
7	F6	0	1	2	1.5	72.8
5	F7	1	1	3	1.5	66.5
8	F8	0	0	2	1	69.5
10	F9	0	0	2	1	71.2

* F5 was the center points in the design and repeated three times.

After liposome formation, the free PL-5 peptide not encapsulated within the liposomes was separated from the liposomal suspension by ultracentrifugation at 100,000 × g for 30 min [28]. The supernatant containing the free peptide was removed, and the liposome pellet was resuspended in PBS (pH 7.4). This step ensures that only the peptide encapsulated in the liposomes is measured for further analysis.

### 2.3. Formulation optimization

To optimize the liposome formulation for PL-5 encapsulation, a 3-level factorial response surface method (RSM) with Miscellaneous was employed using Design-Expert 8.0.5b software (Stat-Ease Inc., Minneapolis, USA), following established protocols for experimental design [29]. The two independent variables, molar ratio of DSPC/Chol (X_1_) and molar ratio of DSPE-PEG_2000_/DSPC (X_2_), were selected based on preliminary studies, and their effects on the encapsulation efficiency (EE) of PL-5-loaded liposomes were investigated. The molar ratio of PoP/Chol/DOTAP was fixed at 1:2:1 throughout all formulations to ensure optimal photodynamic responsiveness while preserving liposomal membrane stability.

The independent variables were varied at three levels: low (–1), medium (0), and high (+1), resulting in nine distinct experimental runs. The center point (F5) was repeated three times to assess the reproducibility of the formulations. The EE of the liposomes (dependent variable Y) was measured for each formulation, as detailed in Table 1.

The experimental data were analyzed using Design-Expert 8.0.5b software (Stat-Ease, Inc.), and statistical analysis was performed using Analysis of Variance (ANOVA) to evaluate the significance of the effects of independent variables on encapsulation efficiency.

### 2.4. Size and zeta potential

The mean diameter and polydispersity index (PDI) of the liposomes were measured by dynamic light scattering (DLS) using a Malvern Zetasizer Pro (Malvern Panalytical Ltd) at 25°C. The zeta potential of the liposomes was also determined by DLS to assess the surface charge, which impacts the stability of the liposomes in suspension and their interaction with the cell membranes. DLS measurements were performed at a scattering angle of 90° using disposable polystyrene cuvettes. Prior to measurement, samples were diluted in filtered phosphate-buffered saline (PBS, pH 7.4) to minimize multiple scattering effects and ensure accurate size determination.

### 2.5. Morphology

The morphology of the liposomes was examined by Transmission electron microscopy (TEM). A small aliquot of the liposome suspension was placed on a copper grid and negatively stained with 1% phosphotungstic acid for 1 min. After removing excess stain, the samples were dried and observed under a JEOL-2100F TEM (JEOL Ltd., Japan) at 120 kV. This technique provides detailed visualization of the liposomal structure and confirms the uniformity of the liposome size and shape. For each sample, ten randomly selected fields were imaged using TEM, and the particle diameters were recorded. The average particle diameter was then calculated.

### 2.6. Encapsulation efficiency

The encapsulation efficiency (EE) of PL-5 was determined by quantifying the amount of unencapsulated peptide remaining in the supernatant following ultracentrifugation as described previously. The concentration of free PL-5 was measured using an Agilent 1260 HPLC system equipped with an Agilent Zorbax SB-C18 column (4.6 × 250 mm, 5 μm). Peptides were eluted with a 10−90% acetonitrile gradient in 0.1% trifluoroacetic acid at a flow rate of 1 mL/min, and detected at 224 nm. The injection volume was 10 μL and the retention time of PL-5 was about 8 min. The HPLC method used for PL-5 quantification was validated for linearity (R^2^ > 0.999), precision (relative standard deviation, RSD < 2%), and accuracy (recovery between 98% and 102%).

Encapsulation efficiency was calculated as follows (Eq. 1):


$$\begin{array}{c} \text{Encapsulation Efficiency }(\%)=\text{ Amount of PL}-\text{5}\text{ encapsulated}/\text{Total amount of PL}-\text{5}\text{ initially used}\times \text{100}\% \end{array}$$ (1)

### 2.7. Stability studies

The stability of the PL-5 loaded liposomes was assessed by measuring PL-5 retention in various physiological fluids over incubation periods of 0, 2, 4, 6, 8, 12, 24, and 48 h. Liposomal samples (1 mL) were washed with PBS (pH 7.4) and resuspended in 100 mL of PBS (pH 7.4), simulated wound fluid (SWF A) prepared as Svensby et al. reported [30] or human plasma in triplicate. Suspensions were incubated with gentle agitation (150 rpm) at either 4°C for samples in PBS or at 37°C for samples in PBS, BAL, plasma, or sputum. At each designated time point, samples (1 mL) were collected and centrifuged at 16,000 × g for 15 min at 4°C [31]. The resulting supernatants were analyzed for PL-5 release using an HPLC as above described. Data were obtained from three independent experiments, each performed in duplicate. PL-5 retention (%) was calculated using the following formula (Eq. 2):


$$\begin{array}{cc} \text{PL}-\text{5}\text{ Retention }(\%)= & \;(\text{Concentration of encapsulated }-\text{ Concentration of released})/\\ & \text{Concentration of encapsulated}\times \text{100}\% \end{array}$$ (2)

The long-term stability of the PL-5-loaded liposomes was assessed over a 4-week period at 4°C. The liposomes were periodically analyzed for changes in size, PDI, and encapsulation efficiency to ensure formulation stability.

### 2.8. In vitro evaluation of photodynamic responsiveness

The controlled release of PL-5 from the liposomes was studied under simulated physiological conditions (PBS, pH 7.4, 37°C). A 4 mL portion of liposomes was added to centrifuge tubes and irradiated with red light (λ = 635 nm, 192 J/cm^2^) with a laser power density of 200 mW/cm^2^. The irradiation duration ranged from 0 min to 16 min (192 J/cm^2^) [32]. The amount of released PL-5 was determined by HPLC after ultracentrifugation (100,000 × g, 30 min). The release kinetics were analyzed, and the cumulative release was plotted as a function of time.

### 2.9. Minimum inhibitory concentration (MIC) and minimum bactericidal concentration (MBC)

The antimicrobial activity of empty liposomes, free PL-5 and PL-5 loaded liposomes, with or without red-light irradiation, was evaluated using the broth microdilution method in accordance with Clinical and Laboratory Standards Institute (CLSI) guidelines. Serial dilutions of each formulation were prepared in Mueller-Hinton Broth (MHB). Bacterial inocula were obtained from overnight cultures, adjusted to a 0.5 McFarland standard, and diluted to 5 × 10^5^ colony-forming units (CFU)/mL in wells. Free or liposomal PL-5 was diluted to final concentrations ranging from 0.125 to 64 mg/L (empty liposomes contained no PL-5 but with the same liposomal concentration as corresponding PL-5 loaded liposomes). Empty liposomes in MHB served as formulation controls. For photodynamic activation, PL-5 liposome wells were irradiated at 635 nm to a fluence of 192 J/cm² after 30 min of incubation. Plates were incubated at 37°C for a total of 24 h. The MIC was defined as the lowest concentration preventing visible growth, corresponding to ≥80% reduction in OD_600_ relative to untreated controls. Growth controls (MHB with inoculum) and sterility controls (MHB without inoculum) were included in every assay. Minimum bactericidal concentration (MBC) values were determined by subculturing 10 μL from wells without visible growth onto Mueller-Hinton agar, followed by 24 h incubation at 37°C. The MBC was defined as the lowest concentration that resulted in ≥99.9% (a 3-log_10_) reduction in the original bacterial inoculum [30]. All experiments were conducted in triplicate, each in duplicate.

### 2.10. Time-kill assay

Time-kill studies were performed to evaluate the bactericidal kinetics of free PL-5, PL-5 loaded liposomes (with or without red-light irradiation) at a concentration of one, two, or four times of the MICs. MRSA and MSSA overnight cultures were adjusted to 0.5 McFarland standard and diluted in MHB to 1 × 10^6^ CFU/mL, which yielded a starting density of approximately 5 × 10^5^ CFU/mL after the addition of the formulations. Bacterial suspensions were incubated with each formulation at 37°C. For the red-light group, PL-5 loaded liposomes were irradiated with red light (635 nm, 192 J/cm²) after 30 min of incubation. Samples were collected at 2, 4, 6, 8, 12, and 24 h. Each sample was serially diluted and plated in triplicate onto Mueller-Hinton Agar. After 18–24 h incubation at 37°C, CFU were counted from plates with 30–300 colonies. The log_10_ CFU/mL from three independent experiments was plotted over time to generate time-kill curves [33].

### 2.11. Biofilm formation and inhibition

To assess the biofilm inhibition activity, the effect of formulations on initial biofilm formation by *S. aureus* (MRSA and MSSA) was evaluated. Overnight cultures of *S. aureus* isolates were adjusted to 0.5 McFarland and diluted to 1 × 10^6^ CFU/mL in MHB. In sterile 96-well plates, 100 μL of bacterial suspension was combined with 100 μL of free or PL-5-loaded liposomes (0.125–64 mg/L). Growth and sterility controls were included. For the red-light group, PL-5 liposome wells were irradiated at 635 nm (192 J/cm²) after 30 min incubation. Plates were incubated at 37°C for 24 h, washed with PBS, and biofilms fixed with methanol, stained with 0.1% crystal violet, and solubilized in 33% acetic acid. Biofilm biomass was measured at OD_590_, and inhibition (%) calculated relative to untreated controls [31]. Experiments were performed in triplicate with duplicate wells.

### 2.12. Statistical analysis

All experiments were conducted in triplicate (n = 3, based on preliminary experimental variability), and results are expressed as mean ± standard deviation (SD). ANOVA followed by Tukey’s post-hoc test was used to compare multiple groups. A significance level of p < 0.05 was considered statistically significant. Data analysis was conducted using IBM SPSS Statistics 23.0 software.

## 3. Results

### 3.1. Formulation optimization

The Design-Expert software suggested that a quadratic model best fit the data for EE, with highly significant p-values (less than 0.0001). ANOVA and regression analysis further confirmed the statistical significance of the model, as shown in Table 2. The Model F-value of 86.15 indicates that the model is highly significant, with only a 0.01% chance of this F-value occurring due to random noise. P-values less than 0.05 indicated that the terms X_1_ (DSPC/Chol), X_2_ (DSPE-PEG_2000_/DSPC), and X_1_^2^ were significant factors in the model. The Lack of Fit F-value of 0.44 indicated that the lack of fit was not significant relative to the pure error, with a 75.07% chance of the lack of fit occurring due to noise, which is acceptable.

**Table 2:** Fit summary and ANOVA of the quadratic model.

Source	Sum of Squares	df	Mean Square	F-value	p-value
**Model**	206.39	5	41.28	86.15	< 0.0001
X_1_-DSPC/Chol	5.80	1	5.80	12.11	0.0177
X_2_-DSPE-PEG2000/DSPC	54.60	1	54.60	113.96	0.0001
X_1_ X_2_	0.0625	1	0.0625	0.1304	0.7327
X_1_²	128.56	1	128.56	268.31	< 0.0001
X_2_²	0.9854	1	0.9854	2.06	0.2110
**Residual**	2.40	5	0.4791		
Lack of Fit	0.9491	3	0.3164	0.4374	0.7507
Pure Error	1.45	2	0.7233		
**Cor Total**	208.79	10			

The polynomial equation derived for the dependent variable (EE) was as follows:


$$\begin{array}{c} \text{Y}=\text{31}.\text{00}+\text{29}.\text{73}*{\text{X}}_{\text{1}}+\text{11}.\text{52}*{\text{X}}_{\text{2}}-\text{0}.\text{25}*{\text{X}}_{\text{1}}*{\text{X}}_{\text{2}}-\text{7}.\text{12}*{\text{X}}_{\text{1}}*{\text{X}}_{\text{1}}-\text{02}.\text{49}*{\text{X}}_{\text{2}}*{\text{X}}_{\text{2}} \end{array}$$ (3)

**Fig 1:**
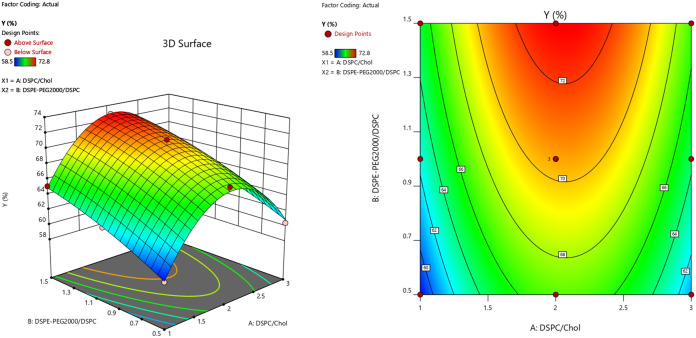
The influence of the independent variables on encapsulation efficiency (Y) in 3D response surface plot.

Fig 1 shows a graphical representation of the effects of the independent variables on EE across all formulations. The 3D surface plots illustrate how varying lipid ratios impact PL-5 encapsulation, with optimal conditions marked for maximum EE. After inputting the “maximum EE” target into the software, it generated an optimized formulation of DSPC/Chol with 2 and DSPE-PEG_2000_/DSPC with 1.5, which was similar to the formulation of F6 in the Table 1. Therefore, F6 was selected as the optimized batch for further evaluation.

The optimized formulation (F6) yielded a predicted EE% of 73.1%. Experimental validation produced an EE% of 72.8 ± 2.4% (n = 3), indicating a good agreement between the predicted and experimental values. The drug loading of the optimized formulation was 4.2 ± 0.3% (w/w).

### 3.2. Size, polydispersity index (PDI), and zeta potential

**Fig 2:**
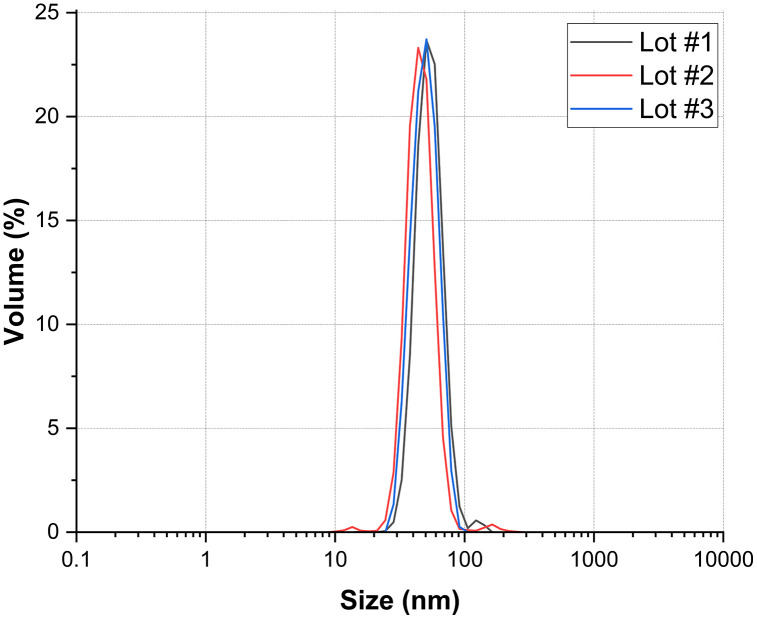
Dynamic light scattering (DLS) graph of the size distribution of the PL-5 loaded liposomes (n = 3).

The mean diameter of the optimized liposomes (F6) was 50 ± 6 nm (Fig 2), with a polydispersity index (PDI) of 0.24, indicating a narrow size distribution (Although a PDI < 0.2 is ideal, a value of 0.24 is still within acceptable limits for nanomedicine formulations and reflects the homogeneity achieved after extrusion), which is ideal for consistent drug delivery and efficient penetration through biological barriers, including biofilms. The zeta potential of the liposomes was + 10.3 ± 1.1 mV (S1 Appendix), suggesting good stability due to electrostatic repulsion between particles, which prevents aggregation.

### 3.3. Morphological characterization

**Fig 3:**
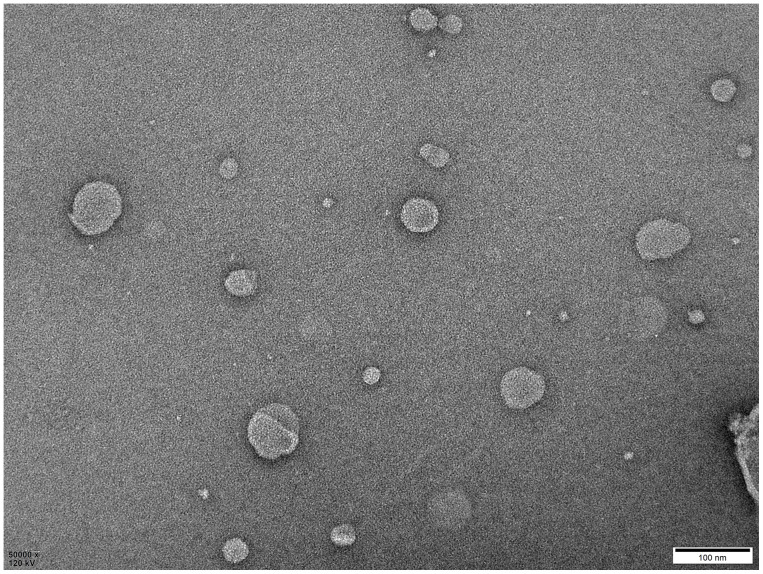
Transmission electron microscopy (TEM) images of the PL-5 loaded liposomes.

TEM images revealed that the liposomes had a spherical morphology with well-defined membranes (Fig 3), consistent with the formation of stable, uniform liposomes. The size observed by TEM (48 ± 7 nm, n = 50 particles) was in good agreement with the DLS data.

### 3.4. In vitro stability

**Fig 4:**
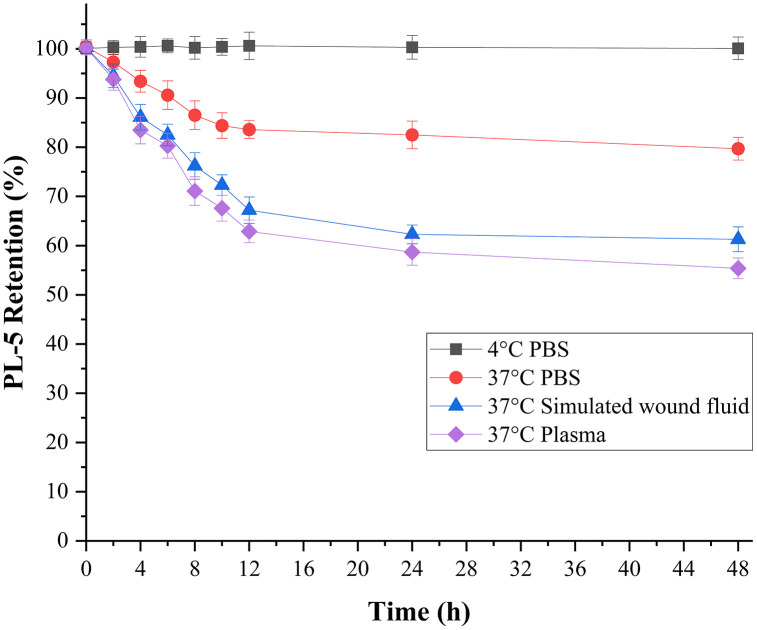
Stability of PL-5-loaded liposomes in pH 7.4 phosphate buffer saline (PBS) at 4°C, in PBS at 37°C, simulated wound fluid at 37°C and plasma 37°C. Data represent mean ± SD from three independent experiments (n = 3).

The stability of PL-5 loaded liposomes was evaluated by quantifying PL-5 retention following incubation in PBS, simulated wound fluid or plasma over a 48-h period. PL-5 retention remained unchanged in PBS at 4°C, maintaining values near 100% for the entire duration (Fig 4), indicating negligible PL-5 leakage under refrigerated storage conditions. At 37°C in PBS, a gradual decline in PL-5 retention was observed, decreasing from 100.4% at 0 h to 79.7% at 48 h. Release was more pronounced in biological fluids. In simulated wound fluid, PL-5 retention dropped rapidly during the first 12 h, decreasing from 100.3% to 67.2%, and further to 61.3% by 48 h. Plasma induced the greatest PL-5 release among the biological fluids tested, leading to a decline from 100.2% at 0 h to 55.4% at 48 h. Across all fluids, differences in retention emerged as early as 2 h and progressed steadily throughout the incubation period. Overall, the liposomes showed strong stability in PBS at 4°C, moderate stability in PBS at 37°C, and reduced stability in simulated wound fluid and plasma. These findings suggest that the extent of PL-5 release is strongly influenced by the biochemical complexity and enzyme content of the surrounding milieu.

The long-term *in vitro* stability of PL-5 loaded liposomes was assessed over a period of 4 weeks at 4°C. The liposomes showed excellent stability, with only slight variations in size (±10%), zeta potential (±7%) and a minimal decrease in EE (from 72.3% to 70.8%) during storage. These results suggest that the formulation was stable over time of 4 weeks, making it suitable for potential clinical applications.

### 3.5. Photodynamic response peptide release

**Fig 5:**
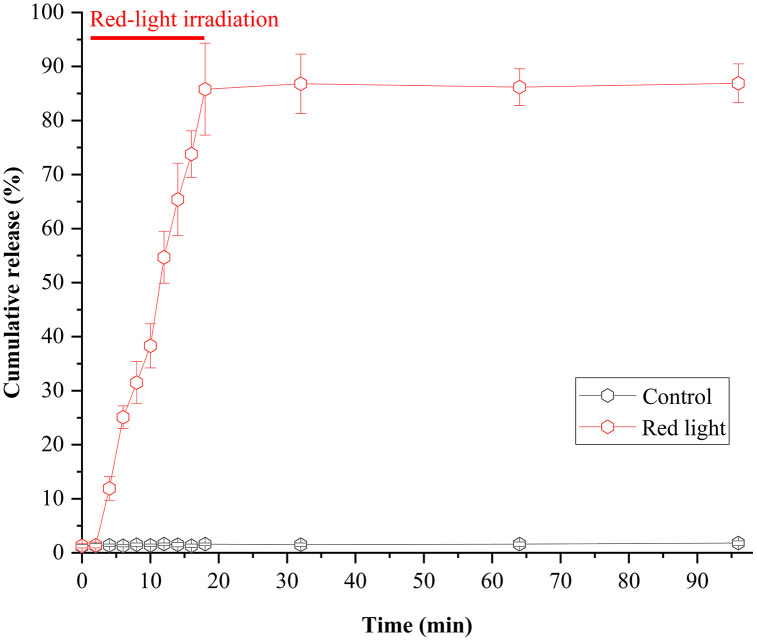
The cumulative release of PL-5 from the liposomes under red-light exposure (λ = 635 nm, 192 J/cm^2^). Data represent mean ± SD from three independent experiments (n = 3).

The *in vitro* evaluation of PL-5 releases from the liposomes under red light (λ = 635 nm) irradiation revealed significant responsiveness to light exposure. As shown in Fig 5, liposomes exhibited a rapid release of PL-5 upon exposure to red light (λ = 635 nm, 192 J/cm², 200 mW/cm²). The cumulative release of PL-5 increased progressively with the irradiation time, reaching over 85% release after 16 min of irradiation. This release profile highlights the light-triggered mechanism of action enabled by the PoP component of the liposomes. In the absence of light (control group), the release of PL-5 was minimal, with less than 2% release observed across the time points (0–96 min). In contrast, when exposed to red light, the release of PL-5 significantly accelerated, as evidenced by the rapid increase in the percentage of released drug. Notably, after 4 min of light exposure, approximately 25% of the encapsulated PL-5 was released, and by 14 min, over 70% of the payload had been liberated from the liposomes. After the light irradiation, there was less than 1% peptide release increase. This data demonstrates that the liposomal system exhibits a robust and controlled release profile that is highly responsive to light, which aligns with previously reported findings on light-triggered drug release from liposomes [32].

### 3.6. Antimicrobial activity

The antimicrobial activities of free PL-5 and PL-5-loaded liposomes, with or without red-light irradiation, were assessed by MICs and MBCs against MRSA and MSSA isolates (Table 3). Empty liposomes (with or without red light) showed no detectable antimicrobial activity (MIC and MBC > 128 mg/L), and their values differed significantly from all PL-5-containing formulations (p = 0.006). Free PL-5 exhibited moderate antibacterial activity against both strains, with MIC/MBC values of 12/24 mg/L for MRSA and 8/16 mg/L for MSSA. Exposure to red light did not significantly alter the MIC or MBC values of free PL-5 (p = 0.17), indicating that irradiation alone had no measurable effect on peptide antimicrobial activity.

**Table 3:** MIC and MBC values of free PL-5 and PL-5 loaded liposomes with or without red-light irradiation.

Formulation	MIC MRSA (mg/L)	MBC MRSA (mg/L)	MIC MSSA (mg/L)	MBC MSSA (mg/L)
Empty liposomes	>128	>128	>128	>128
Empty liposomes (red light)	>128	>128	>128	>128
Free PL-5	12	24	8	16
Free PL-5 (red light)	12	24	8	16
PL-5 liposomes	32	64	32	48
PL-5 liposomes (red light)	8	20	4	8

Non-irradiated PL-5-loaded liposomes showed significantly reduced antibacterial efficacy compared with free PL-5, with MIC and MBC values of 32 and 48 mg/L, respectively, against both MRSA and MSSA (p = 0.02). In contrast, red-light–irradiated PL-5-loaded liposomes (192 J/cm²) exhibited significantly enhanced antimicrobial activity relative to their non-irradiated counterparts (p = 0.006). Upon irradiation, MIC values decreased fourfold, reaching 8 mg/L for MRSA and 4 mg/L for MSSA, while MBC values were reduced to 20 mg/L and 8 mg/L, respectively (p = 0.004).

Furthermore, red-light-activated PL-5-loaded liposomes demonstrated significantly lower MIC and MBC values than free PL-5 for both MRSA and MSSA (p = 0.02), indicating superior inhibitory and bactericidal activity following photodynamic activation.

Collectively, these results indicate that red-light irradiation significantly potentiates the antimicrobial activity of PL-5 when delivered via liposomes, leading to statistically significant reductions in both inhibitory and bactericidal concentrations against MRSA and MSSA, whereas free PL-5 remains unaffected by light exposure.

### 3.7. Time-kill kinetics

**Fig 6:**
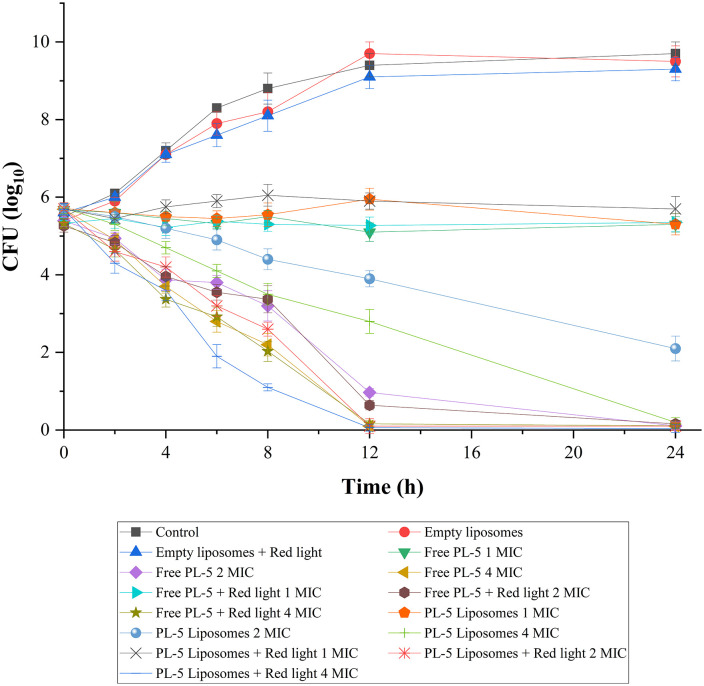
Killing curve of methicillin-resistant *S. aureus* (MRSA) strain exposed to 1× , 2× , and 4× MIC of PL-5. Red-light-activated PL-5 liposomes exhibited the most rapid and extensive bactericidal activity, achieving ≥3-log₁₀ reduction within 6–8 h at 2 × MIC and complete killing by 12 h. At 4 × MIC, bacterial counts fell below the detection limit within 8–12 h. In contrast, non-irradiated liposomes showed delayed or incomplete killing, and free PL-5 required up to 24 h to achieve comparable reductions. Empty liposomes (with or without light) showed no bactericidal effect. Data represent mean ± SD from three independent experiments (n = 3). Abbreviations: CFU, colony forming unit; MIC, minimum inhibitory concentration.

**Fig 7:**
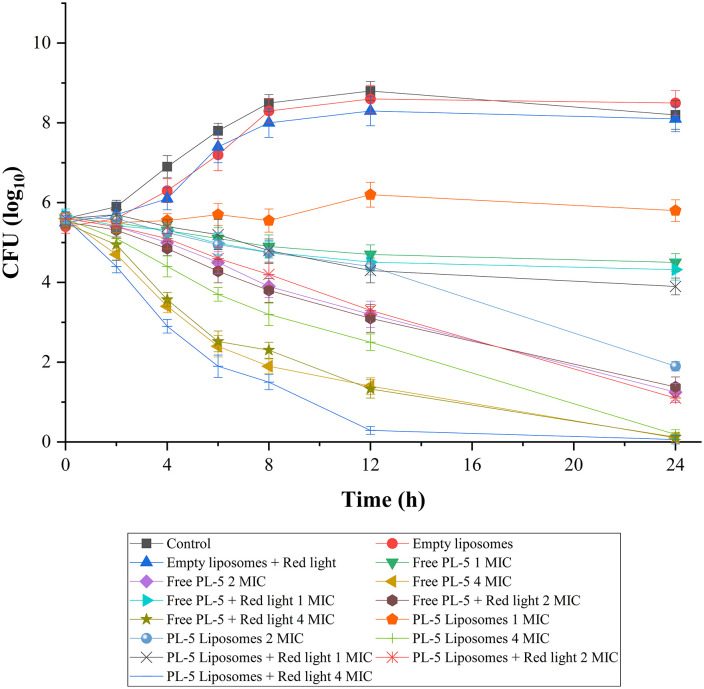
Killing curve of methicillin-susceptible *S. aureus* (MSSA) strain exposed to 1× , 2× , and 4× MIC of PL-5. Red-light-activated PL-5 liposomes demonstrated the fastest and most potent killing, with ≥3-log₁₀ reduction observed within 4–6 h at 2 × MIC and complete eradication by 12 h. At 4 × MIC, bacterial counts fell below the detection limit within 8 h. Free PL-5 showed concentration-dependent but slower killing, while non-irradiated liposomes exhibited limited activity, especially at lower concentrations. Empty liposomes (with or without light) had no effect on bacterial viability. Data represent mean ± SD from three independent experiments (n = 3). Abbreviations: CFU, colony forming unit; MIC, minimum inhibitory concentration.

To further characterize bactericidal dynamics, time-kill assays were performed at 1 × , 2 × , and 4 × MIC, as determined for each formulation, against MRSA and MSSA (Figs 6 and 7). Across both strains, empty liposomes (with or without red light) showed no bactericidal kinetics.

Free PL-5 demonstrated concentration-dependent killing but slower bactericidal kinetics. At 2 × MIC, free PL-5 produced gradual reductions in viable counts, reaching ≥3-log_10_ reductions only at later time points (24 h), whereas at 4 × MIC, near-complete killing was observed by 12−24 h. When free PL-5 was combined with red-light exposure, a modest acceleration of bactericidal activity was observed (p = 0.62). Red light slightly enhanced the killing kinetics at all concentrations, but reductions in bacterial counts remained slower than those achieved by light-activated PL-5-loaded liposomes (all p values larger than 0.05).

Non-irradiated PL-5-loaded liposomes showed limited bactericidal activity at 1 × MIC and delayed killing at higher concentrations. Although reductions in bacterial burden were observed at 2× and 4 × MIC, complete sterilization was generally achieved later than with free PL-5, and evidence of bacterial persistence or delayed regrowth was observed, particularly at lower concentrations.

In contrast, red-light–activated PL-5-loaded liposomes exhibited the most rapid and extensive bactericidal activity against both MRSA and MSSA. At 2 × MIC, activated liposomes induced ≥3-log_10_ reductions within 6−8 h and achieved complete killing by 12 h for both strains. At 4 × MIC, bactericidal effects were further accelerated, with ≥3-log_10_ reductions occurring as early as 4 h and bacterial counts declining to near or below the limit of detection by 8−12 h. At corresponding time points and concentrations, activated liposomes achieved significantly greater reductions in CFU compared with free PL-5 and non-irradiated liposomes (p = 0.03).

Overall, the combined MIC/MBC and time-kill data demonstrate that red-light activation consistently enhances the antibacterial efficacy of PL-5-loaded liposomes against both MRSA and MSSA. This enhancement is characterized by reduced inhibitory and bactericidal concentrations, faster killing kinetics, and sustained bactericidal activity, supporting the role of light-triggered liposomal permeabilization in improving antimicrobial performance.

### 3.8. Biofilm inhibition assay

**Fig 8:**
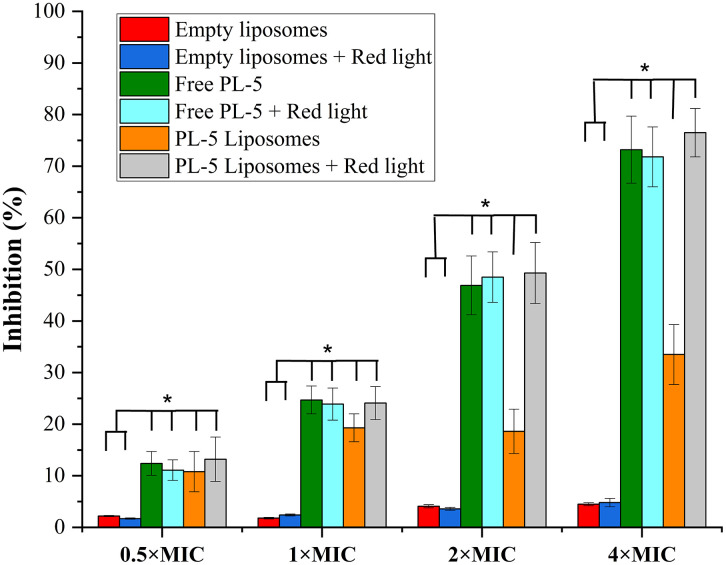
Biofilm inhibition of methicillin-resistant *S. aureus* (MRSA) by PL-5 formulations. Free PL-5 exhibited concentration-dependent inhibition, reaching 73.2 ± 6.5% at 4 × MIC. Non-irradiated PL-5 liposomes showed limited antibiofilm activity (≤33.5 ± 5.8% at 4 × MIC). Red-light activation significantly enhanced the efficacy of PL-5 liposomes, achieving inhibition comparable to free PL-5 (76.5 ± 4.7% at 4 × MIC). Empty liposomes (with or without light) showed negligible inhibition (<6%). *P < 0.05, data represent mean ± SD from three independent experiments (n = 3). Abbreviations: MIC, minimum inhibitory concentration*.*

**Fig 9:**
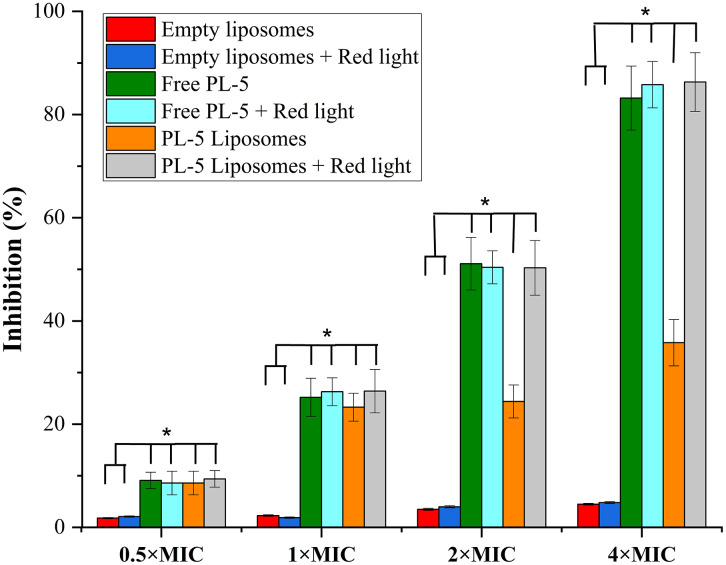
Biofilm inhibition of methicillin-susceptible *S. aureus* (MSSA) by PL-5 formulations. Free PL-5 caused concentration-dependent inhibition, with up to 83.2 ± 6.2% reduction at 4 × MIC. Non-irradiated PL-5 liposomes displayed weak antibiofilm effects (≤35.8 ± 4.5% at 4 × MIC). Red-light activation markedly improved the performance of PL-5 liposomes, yielding inhibition of 86.3 ± 5.7% at 4 × MIC, comparable to free PL-5. Empty liposomes (with or without light) showed negligible inhibition (<6%). *P < 0.05, data represent mean ± SD from three independent experiments (n = 3). Abbreviations: MIC, minimum inhibitory concentration*.*

The antibiofilm activities of free PL-5, PL-5–loaded liposomes, and red-light–activated PL-5–loaded liposomes against MRSA and MSSA were evaluated using crystal violet staining after 24 h incubation (Figs 8 and 9). Empty liposomes, with or without red-light irradiation, showed negligible biofilm inhibition (<6%) at all tested concentrations.

Free PL-5 exhibited a clear concentration-dependent inhibition of biofilm formation for both MRSA and MSSA. In MRSA, biofilm inhibition increased from 12.4 ± 2.3% at 0.5 × MIC to 73.2 ± 6.5% at 4 × MIC. Similarly, for MSSA, inhibition ranged from 9.1 ± 1.6% at 0.5 × MIC to 83.2 ± 6.2% at 4 × MIC, indicating a higher susceptibility of MSSA biofilms to PL-5 at elevated concentrations.

In contrast, non-irradiated PL-5 loaded liposomes produced only modest antibiofilm effects. For MRSA, inhibition ranged from 10.8 ± 3.9% at 0.5 × MIC to 33.5 ± 5.8% at 4 × MIC, while for MSSA inhibition ranged from 8.6 ± 2.3% to 35.8 ± 4.5% over the same concentration range. These values were consistently lower than those achieved with free PL-5 at corresponding concentrations (p = 0.02), suggesting limited PL-5 availability in the absence of photoactivation.

Red-light activation markedly enhanced the antibiofilm efficacy of PL-5 loaded liposomes. For MRSA, biofilm inhibition reached 49.3 ± 5.9% at 2 × MIC and 76.5 ± 4.7% at 4 × MIC, significantly exceeding the effects of non-irradiated liposomes (p = 0.03). A similar enhancement was observed for MSSA, with inhibition of 50.3 ± 5.3% at 2 × MIC and 86.3 ± 5.7% at 4 × MIC. At concentrations ≥1 × MIC, red-light activated liposomes achieved antibiofilm activity comparable to that of free PL-5.

Overall, these findings demonstrate that photodynamic activation substantially potentiates the antibiofilm activity of PL-5 when delivered via liposomes, effectively restoring or matching the efficacy of free PL-5 against both MRSA and MSSA biofilms.

## 4. Discussion

The liposome formulation was composed of DSPC as the primary phospholipid to ensure membrane stability, while Chol was included to enhance membrane integrity. PoP facilitated red-light responsive release, and DOTAP provided positive charges to promote bacterial interaction, while also reducing peptide-liposome interaction through electrostatic repulsion. DSPE-PEG_2000_ contributed steric stabilization, minimizing the interaction between PL-5 and the liposome membrane, reducing aggregation, and prolonging the residence time of the liposomes at the wound site.

The optimization of PL-5-loaded photodynamic-responsive cationic liposomes revealed that the molar ratio of DSPC/Chol (X_1_) and DSPE-PEG_2000_/DSPC (X_2_) play crucial roles in determining the EE of the formulation. According to the results derived from the quadratic equation (Equation 1), both X_1_ and X_2_ showed a positive correlation with EE, meaning that increasing the ratios of DSPC and DSPE-PEG_2000_ improved the encapsulation efficiency. This can be attributed to the stabilization of the liposomal membrane, which enhances the encapsulation and retention of PL-5 within the liposomes. Increasing the amount of DSPE-PEG_2000_ in the formulation likely contributes to a more stable and sterically stabilized liposomal structure, which prevents premature release of the encapsulated drug. These findings align with previous studies showing that liposomal formulations with increased PEGylation typically exhibit enhanced stability and improved drug retention [34]. DSPC, being a phospholipid with higher gel transition temperatures, is likely to form more robust bilayers at elevated ratios, leading to improved encapsulation [35]. These results suggesting that liposomal formulations with higher phospholipid content and PEGylation have improved drug encapsulation and delivery efficiency. Moreover, response surface plots (Fig 1) further elucidated the relationship between the formulation variables and EE, showing a clear positive impact of X_1_ and X_2_ on liposomal encapsulation. The 3D surface plots indicated optimal regions for maximum EE, which were further confirmed through the experimental results of F6, identified as the optimized formulation.

The optimized PL-5-loaded liposomes in this study exhibited physicochemical properties that are conducive to effective antimicrobial delivery. The mean particle size of ~50 nm and narrow polydispersity index (PDI = 0.24) observed by DLS indicate a highly homogeneous nanocarrier population. Nanocarriers with diameters below ~100 nm have been shown to penetrate bacterial biofilms more efficiently than larger particles, facilitating closer contact with biofilm-embedded bacteria and improving localized drug delivery [24,36]. A low PDI also reflects formulation uniformity, which is important for reproducible biological performance and scalable production.

Surface charge is another critical determinant of nanocarrier behavior in biological systems. The positive zeta potential (+10.3 mV) observed for the PL-5 liposomes not only contributes to colloidal stability through electrostatic repulsion, reducing aggregation, but also may enhance interactions with negatively charged bacterial cell surfaces and biofilm matrices. Such electrostatic attraction has been implicated in improved association of cationic nanocarriers with biofilm components, potentially promoting more effective drug release in proximity to microbial targets [36].

Morphological assessment by TEM confirmed that the liposomes possessed well-defined spherical structures consistent with stable bilayer assembly. The agreement between TEM and DLS size measurements suggests minimal aggregation or deformation during the preparation process, a desirable feature for maintaining consistent delivery behavior across experimental and future translational studies.

The *in vitro* stability profile further reflects the interplay between formulation integrity and environmental complexity. Near-complete PL-5 retention in PBS at 4°C over 48 h and minimal change over 4 weeks at 4°C demonstrate excellent storage stability, which is critical for potential clinical translation and shelf-life considerations. At physiological temperature (37°C), gradual release in PBS can be attributed to increased bilayer fluidity, a behavior commonly observed in phospholipid vesicles [37].

The observed light-triggered release of PL-5 from the liposomes is consistent with other studies exploring the use of photosensitive liposomes for controlled drug delivery. Photosensitive liposomes have gained attention in recent years due to their ability to release payloads in a highly controlled manner upon exposure to specific wavelengths of light, offering significant advantages in the treatment of localized diseases, such as wound infections or cancer, where precise drug delivery is critical [38]. The release kinetics observed in this study are comparable to those reported by Enzian et al., who demonstrated that porphyrin-based liposomes could achieve up to 75% release of encapsulated drugs with 635 nm of light exposure, thus providing a rapid and efficient drug delivery mechanism [39].

Antimicrobial activity results demonstrate that red-light activation significantly enhanced the antimicrobial and antibiofilm efficacy of PL-5 when delivered via a light-responsive liposomal system. Free PL-5 exhibited moderate antibacterial activity, whereas non-irradiated PL-5-loaded liposomes showed reduced efficacy, likely due to retention of the peptide within the vesicle and limited immediate availability for bacterial interaction [40]. Upon red-light irradiation, however, PL-5-loaded liposomes demonstrated significantly lower MIC and MBC values and faster bactericidal kinetics compared with both free PL-5 and non-irradiated liposomal formulations against MRSA and MSSA. This enhancement may be partially explained by light-triggered permeabilization of the liposomal membrane, which facilitates rapid PL-5 release at the target site. The resulting localized, transiently high peptide concentrations may contribute to more efficient disruption of bacterial membranes, consistent with the known mechanism of action of cationic antimicrobial peptides [41]. Such on-demand, high-concentration pulses of PL-5 at the bacterial surface may help overcome tolerance mechanisms associated with lower, diffusely distributed peptide levels. These observations are in line with the behavior of stimuli-responsive nanocarriers, where exogenous triggers such as light promote rapid, spatially confined drug release [42].

Time-kill analyses further showed that red-light-activated liposomes produced ≥3log10 reductions in bacterial counts within 4–8 h and achieved near-complete sterilization by 12 h, whereas free PL-5 required higher multiples of MIC and longer incubation times to achieve similar effects. The accelerated kinetics likely result from the combined effects of localized peptide release and enhanced interaction with bacterial cells within the microenvironment. Similar observations have been reported for other stimuli-responsive delivery systems in which on-demand release improves antimicrobial performance in complex bacterial communities [43].

Biofilm inhibition results corroborate these findings, showing that red-light-activated liposomes significantly suppressed biofilm formation at concentrations ≥1 × MIC, whereas non-irradiated liposomes had only modest effects. Biofilm structures impede antibiotic penetration and increase resistance relative to planktonic cells, representing a major clinical challenge [24]. Controlled release strategies that enhance penetration and local drug concentration within biofilms thus offer a promising route to improve treatment outcomes. Future work should be conducted to investigate the permeability and diffusion behavior of activated liposomes through biofilm matrices using advanced imaging techniques and quantitative diffusion models.

The light-activated PL-5 liposomal system shows strong potential for topical treatment of burn wound infections, which are frequently complicated by biofilm formation and multidrug-resistant pathogens. The ability to trigger localized antimicrobial release using externally applied red light enables precise spatiotemporal control, maximizing antibacterial efficacy at the wound site while minimizing systemic exposure. Integration of this approach with existing burn care practices, such as wound dressing changes and debridement, could enhance biofilm disruption and accelerate bacterial clearance. Importantly, red-light sources are clinically accessible and non-invasive, supporting the translational feasibility of this strategy for managing infected burn wounds.

Despite the above promising findings, several limitations should be noted. First, the study utilized simplified *in vitro* planktonic and biofilm models that do not fully recapitulate the heterogeneous and immune-active microenvironment of burn wounds. Second, the penetration depth of red light in tissue is limited, which may pose challenges for treating deeper or extensive infections; optimization of light delivery parameters or alternative wavelengths may be necessary for clinical translation. Third, while the liposomes exhibited good stability under experimental conditions, their structural integrity, PL-5 retention, and performance in enzyme-rich, dynamic wound environments require further validation *in ex vivo* or *in vivo* burn wound models. Future studies will focus on evaluating the therapeutic efficacy and safety of this light-responsive liposomal system in *in vivo* burn wound infection models. These studies will include assessments of biofilm disruption, wound healing progression, and host immune responses following treatment. In addition, further optimization of light delivery parameters (e.g., irradiation intensity, duration, and dosing intervals) will be explored. Alternative wavelengths or delivery approaches that enable deeper tissue penetration may also be investigated to further enhance the clinical applicability of this light-triggered antimicrobial strategy.

## 5. Conclusions

In this study, a red-light-responsive PL-5-loaded liposomal delivery system was successfully designed, optimized, and evaluated for antimicrobial and antibiofilm applications relevant to burn wound infections. The optimized formulation exhibited favorable physicochemical properties, including high encapsulation efficiency, nanoscale size, positive surface charge, and robust stability under storage conditions. Importantly, incorporation of porphyrin-phospholipids enabled precise, on-demand peptide release upon red-light irradiation, while minimizing premature leakage in the absence of light.

Functionally, red-light activation significantly potentiated the antibacterial efficacy of PL-5 against both MRSA and MSSA, as evidenced by reduced MIC and MBC values, faster killing kinetics, and enhanced suppression of biofilm formation. In contrast, free PL-5 and non-irradiated liposomes displayed comparatively limited activity, highlighting the critical role of light-triggered release in maximizing therapeutic performance. Together, these results support the concept that externally controlled liposomal permeabilization can overcome key limitations of AMP therapy, including insufficient biofilm penetration and delayed bactericidal action.

Overall, this work establishes a robust and reproducible platform for light-controlled AMP delivery and underscores its potential for localized management of biofilm-associated burn wound infections. Future studies involving *in vivo* burn models and safety evaluations will be essential to further validate the clinical translational potential of this approach.

## Supporting information

S1 Appendix Zeta potential of the optimized liposomes. (PDF)
