# Supplementary material for: Preparation and in vitro evaluation of photodynamic-responsive nanoliposome loaded PL-5
Source: PLoS One. 2026 Jun 16;21(6):e0351679. doi: 10.1371/journal.pone.0351679 (PMC13271455; doi:10.1371/journal.pone.0351679)
Supplement: S1 Appendix — (PDF) [file pone.0351679.s001.pdf]

Zeta Potential - Quality

Malvern Panalytical

Zeta potential distribution

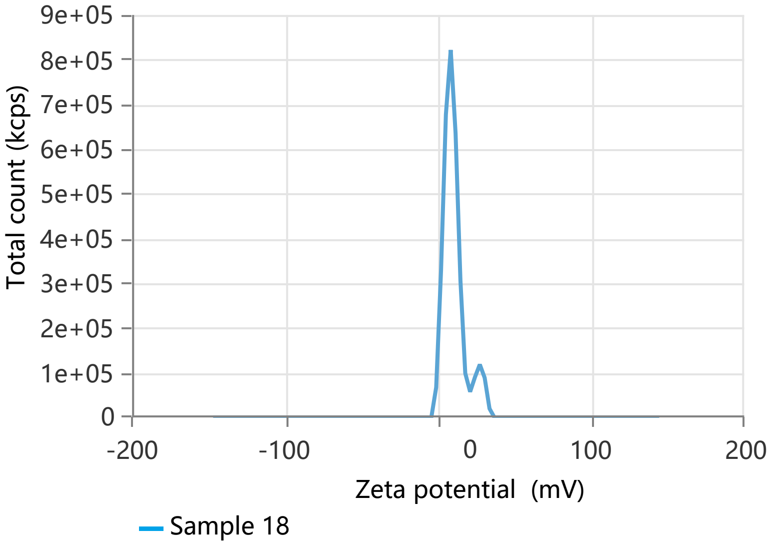

Phase diagram

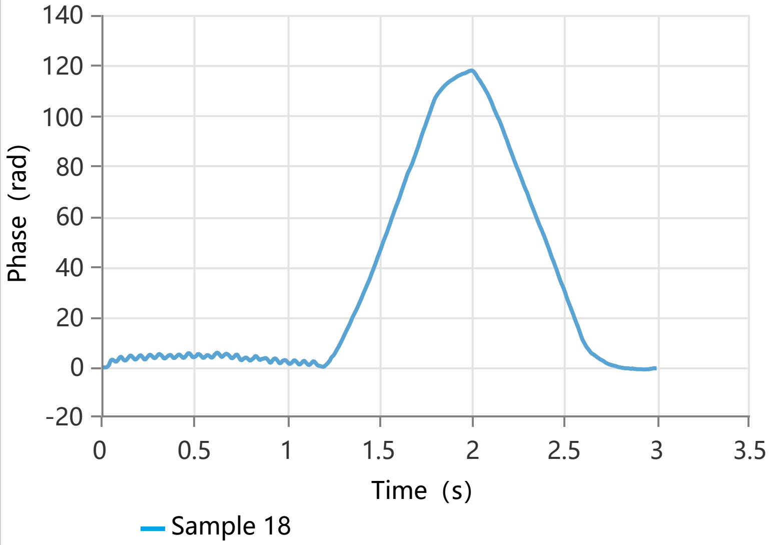

Statistical Table

Name		Mean		Min	Max
Zeta potential (mV)	-	10.32	-	10.32	10.32
Zeta potential of peak 1 (mV)	-	8.489	-	8.489	8.489
Zeta potential of peak 2 (mV)	-	26.6	-	26.6	26.6
Conductivity (mS/cm)	-	0.3753	-	0.3753	0.3753
Pool wall Zeta potential (mV)	-	18.82	-	18.82	18.82
Zeta potential difference (mV)	-	7.172	-	7.172	7.172
Raw average count rate (kcps)	-	8418	-	8418	8418
Reference beam count rate (kcps)	-	3947	-	3947	3947
Quality factor	-	1.458	-	1.458	1.458

List of parameters

Actual Instrument Settings\Instrument Serial Number : MAL1296404

Software Version: 3.3.0.42
